# Sarcomatoid urothelial carcinoma of the urethra with an unusual right ventricular metastasis: a case report and review of literature

**DOI:** 10.3389/fonc.2026.2003381

**Published:** 2026-10-21

**Authors:** Jesús Yaringaño, Javier Calvo-Losada, Gerard Oristrell, María E. Semidey, David Marmolejo, Abrham André Geng-Cahuayme, Marc Simó, Richard Mast, Cristina Suárez, Joan Carles, Rafael Morales-Barrera

**Affiliations:** 1 Medical Oncology Department, Vall d’Hebron University Hospital, Barcelona, Spain; 2 Cardio-Oncology Unit, Cardiology Department, Vall d’Hebron University Hospital, Barcelona, Spain; 3 Pathology Department, Vall d’Hebron University Hospital, Barcelona, Spain; 4 Genitourinary, Central Nervous System and Sarcoma Group, Vall d’Hebron Institute of Oncology, Barcelona, Spain; 5 Radiation Oncology Department, Vall d’Hebron University Hospital, Barcelona, Spain; 6 Nuclear Medicine Department, Vall d’Hebron University Hospital, Barcelona, Spain; 7 Radiology Department, Vall d’Hebron University Hospital, Barcelona, Spain

**Keywords:** cardiac metastasis, enfortumab vedotin, multimodality cardiac imaging, primary urethral carcinoma, sarcomatoid urothelial carcinoma

## Abstract

Primary urethral carcinoma is an uncommon malignancy, and cardiac metastasis represents a rare event (~4% of cases) that poses significant diagnostic challenges, particularly in distinguishing myocardial tumors from intracavitary thrombi. Here we report a 54-year-old male diagnosed with cT4N0M0 sarcomatoid urothelial carcinoma of the bulbar urethra. Following neoadjuvant dose-dense MVAC and radical surgery (ypT2N0, PD-L1 TPS 20%), adjuvant nivolumab was initiated. Six months after surgery, he developed locoregional recurrence and an expanding right ventricular mass. Multimodality cardiac imaging—including contrast echocardiography, FDG PET-CT, cardiac CT, and cardiac MRI—demonstrated a solid myocardial lesion with intracavitary extension and adjacent myocardial infiltration, considered highly suspicious for cardiac metastasis in the context of concurrent extracardiac progression. Histological confirmation was not obtained because of the procedural risk associated with extensive right ventricular involvement. Treatment with enfortumab vedotin yielded relative stability of the cardiac and pulmonary metastases; however, relentless locoregional progression occurred, illustrating interlesional response heterogeneity. The patient subsequently received palliative radiotherapy, and best supportive care before passing away 17 months after initial diagnosis. This case underscores the vital role of multimodal imaging in the differential diagnosis of cardiac masses in oncological patients with concurrent thromboembolic risk and its interpretation in conjunction with the disease course when pathology confirmation cannot be obtained. Furthermore, it highlights the therapeutic complexity and dismal prognosis associated with sarcomatoid histology in advanced urethral carcinoma.

## Introduction

Primary urethral carcinoma is a rare malignancy, accounting for less than 1% of malignancies in Europe, occurring approximately five times more frequently in men than in women (1, 2). Five-year cancer-specific survival rates vary according to the level of invasion, ranging from 68% for early/localized disease (≤T2N0) to 53% for locally advanced stages (≥T3/N+) (3, 4). Prognostic factors include age, tumor stage, grade, anatomical location, and histology, particularly in women (4–6).

Several risk factors have been associated with primary urethral carcinoma. High-risk human papillomavirus (HPV) infection has been implicated in both men and women (7, 8), while lichen sclerosus, previous urethroplasty for urethral stricture, and prostate brachytherapy have been reported predominantly in men (9–11). In women, urethral diverticula are a recognized risk factor for secondary malignancy (12, 13). Urothelial carcinoma is the predominant histological subtype (53–68%), followed by squamous cell carcinoma (16-24%) and adenocarcinoma (9-15%) (1–3).

The molecular landscape of urethral carcinoma remains incompletely characterized, reflecting the rarity and heterogeneity of this disease. In a genomic analysis of 127 patients with advanced or metastatic urethral carcinoma, *PIK3CA* alterations were the most frequent potentially actionable genomic events across histological subtypes, while alterations in *PTEN*, *ERBB2*, *FGFR1-3*, *BRAF*, and *BRCA1/2* were also identified (14). Strong PD-L1 expression and high tumor mutational burden in squamous cell carcinoma suggest potentially greater immunotherapy benefit in that histology (15). In summary, the genomic profile of urethral urothelial carcinoma appears broadly similar to that of bladder urothelial carcinoma, although HPV 16/18 positivity may be more frequent (16).

Given its rarity, prospective evidence guiding the management of urethral carcinoma is limited, and treatment strategies are largely extrapolated from bladder cancer and other histologically related malignancies. Surgery remains the cornerstone of treatment for localized disease, whereas multimodal approaches are generally considered for locally advanced or recurrent disease. In the metastatic setting, current guidelines recommend considering systemic therapies established for bladder cancer (17, 18).

Cardiac metastasis from urethral carcinoma is an uncommon clinical event, present in 3.9% of the cases (19). Individual cases of cardiac metastasis from urethral carcinoma have previously been reported (20, 21), with more recent reports further illustrating the heterogeneous clinical and histological presentation of cardiac involvement by urothelial carcinoma (22–24). These reports include cases involving squamous and sarcomatoid differentiation, highlighting that cardiac dissemination can occur across different histological subtypes. Nevertheless, cardiac metastasis arising from primary urethral carcinoma remains exceptionally uncommon, and the diagnostic distinction from thrombotic disease may be particularly challenging in patients with concurrent thromboembolic risk (25–27).

Here, we report a case of sarcomatoid urothelial carcinoma of the bulbar urethra with a right ventricular metastasis identified through the integration of sequential multimodality cardiac imaging and disease course. The case highlights the clinical decision-making required when cardiac biopsy is considered high risk, and the therapeutic challenges associated with an aggressive and rare histological variant of urethral carcinoma.

## Case presentation

A 54-year-old male, non-smoker and without significant medical history, was diagnosed in October 2024 with cT4N0M0 sarcomatoid urothelial carcinoma of the bulbar urethra following evaluation of irritative urinary symptoms and hematuria (**Figures 1**, **2A**). He received eight cycles of neoadjuvant dose-dense methotrexate, vinblastine, doxorubicin, and cisplatin (ddMVAC) until February 2025 followed by phallectomy, cystoprostatectomy, and Indiana pouch reconstruction in March 2025. Histopathological examination showed high-grade (G3) sarcomatoid urothelial carcinoma with a 5% giant cell component, ypT2ypN0 (0/16 lymph nodes), with venous and lymphovascular invasion and associated urothelial carcinoma *in situ*. Given a PD-L1 tumor proportion score of 20%, adjuvant nivolumab was initiated in May 2025.

**Figure 1:**
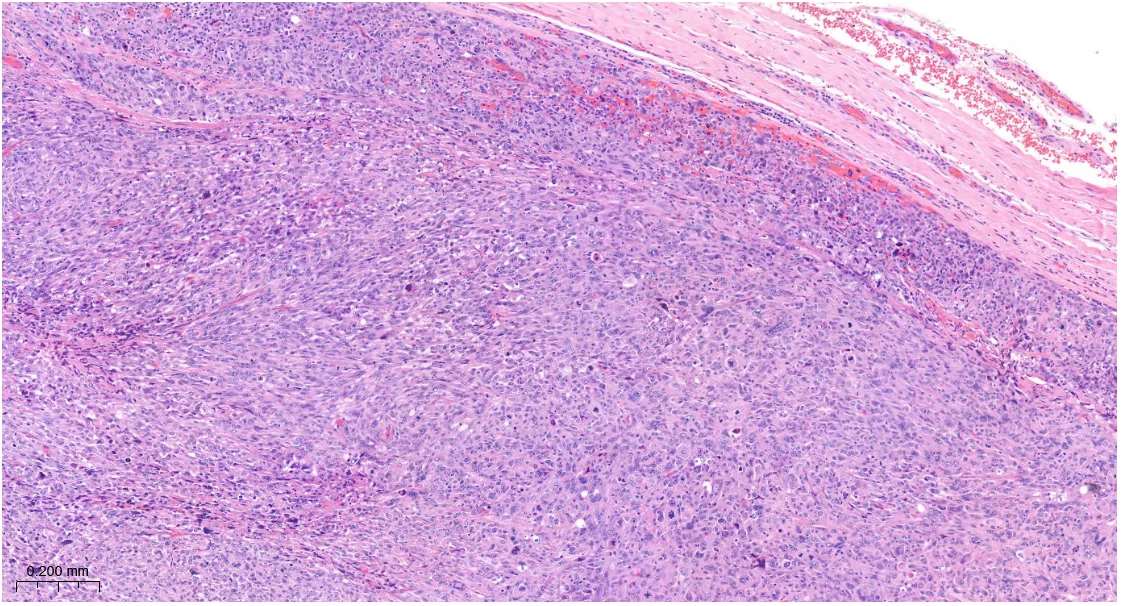
Histopathological appearance of sarcomatoid urothelial carcinoma of the bulbar urethra. Hematoxylin and eosin (H&E) staining. Original magnification 14X; scale bar = 200 µm.

**Figure 2:**
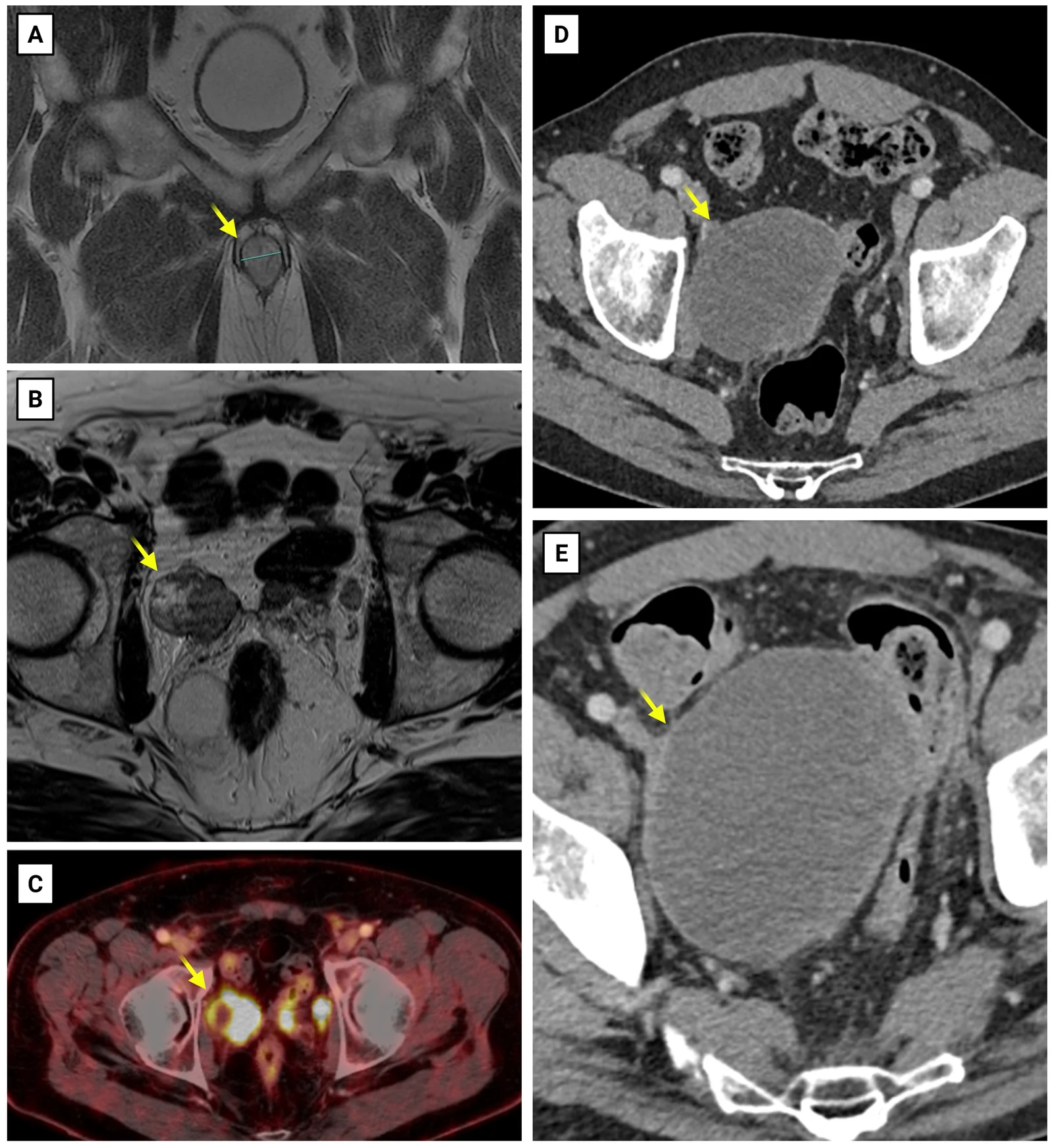
Radiologic evaluation of pelvic disease and locoregional recurrence. **(A)** Initial pelvic MRI (September 2024) showing a 39-mm solid tumor in the bulbar urethra extending into the corpus spongiosum. **(B)** Pelvic MRI (September 2025) demonstrating soft-tissue recurrence in the surgical bed and anterior pelvic peritoneal fascia. **(C)** PET-CT scan (October 2025) shows intense hypermetabolic pelvic disease. **(D)** Abdominal-pelvic CT (December 2025) showing mild progression of pelvic and perineal soft-tissue disease. **(E)** CT scan (January 2026) demonstrating significant locoregional disease progression.

After 3 months of nivolumab treatment, the patient developed fever and left lumbar pain in August 2025. CT urography showed a heterogeneous lesion in the prostatic and right seminal vesicle surgical bed, concerning local recurrence. A filling defect at the right ventricular apex was also identified and initially interpreted as an intracavitary thrombus (**Figure 3A**).

**Figure 3:**
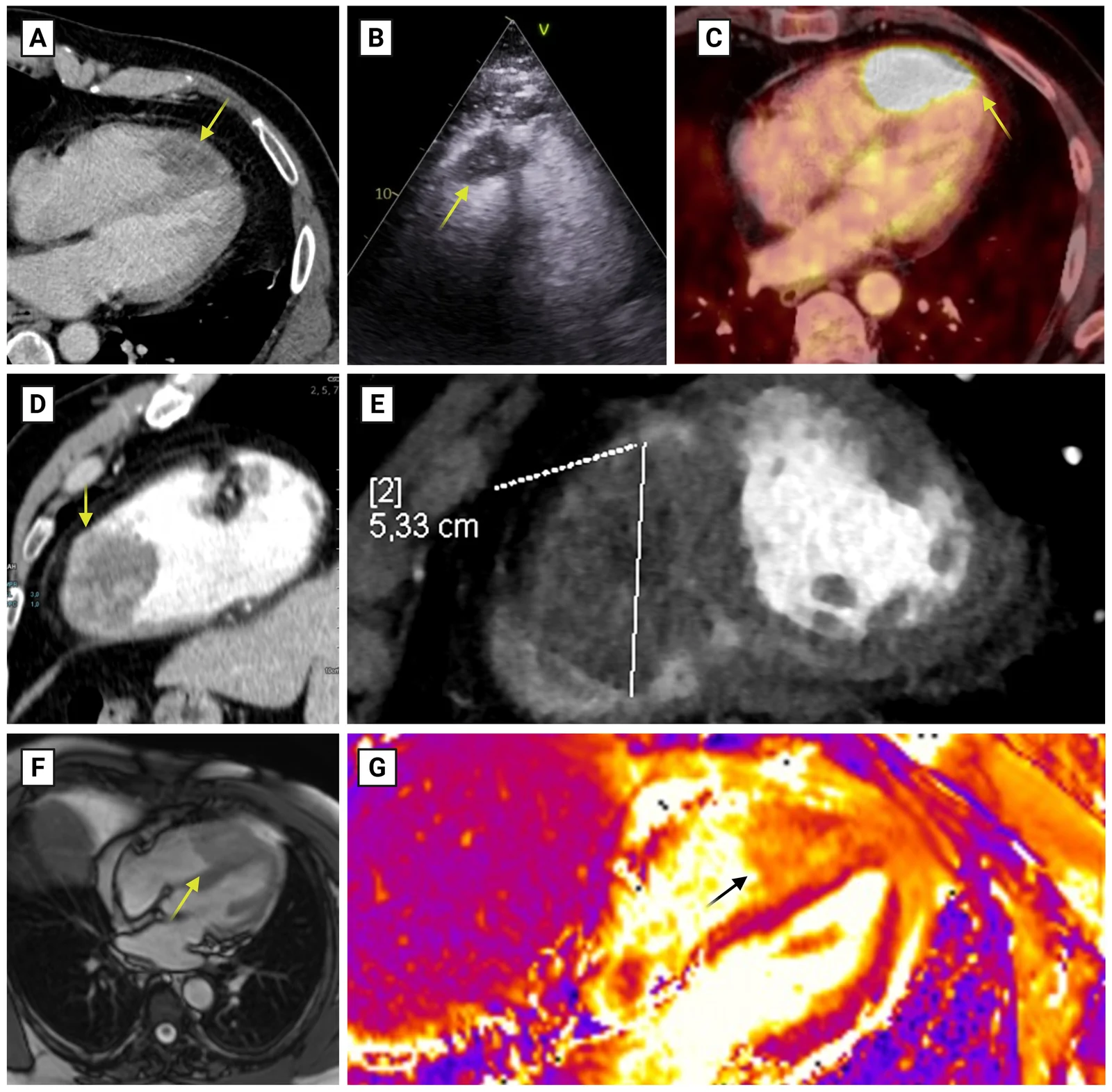
Multimodal cardiac imaging of the right ventricular metastatic mass. **(A)** Contrast-enhanced CT (August 2025) revealing a filling defect at the right ventricular apex, initially interpreted as an intracavitary thrombus. **(B)** Transthoracic echocardiography (August 2025) confirming a 34 × 28 mm apical right ventricular mass. **(C)** PET-CT (October 2025) demonstrates intense FDG uptake within the expanding cardiac lesion (SUVmax 14.9). **(D, E)** Cardiac CT images showing a heterogeneous myocardial mass with peripheral contrast enhancement and central hypodensity measuring 58×53× 38 mm. **(F, G)** Cardiac MRI sequence and T1/T2 mapping illustrating a solid infiltrating lesion arising from the apical septum with late gadolinium enhancement.

Therapeutic anticoagulation with tinzaparin was initiated. Contrast-enhanced transthoracic echocardiography demonstrated mid-apical right ventricular akinesia and a 34x28 mm apical mass, considered compatible with thrombus (**Figure 3B**). CT pulmonary angiography additionally showed a chronic-appearing segmental pulmonary embolus. Lower-extremity Doppler ultrasonography excluded deep vein thrombosis. Following treatment of a concomitant *Klebsiella pneumoniae* urinary tract infection, the patient’s fever and lumbar pain resolved.

Pelvic MRI in September 2025 demonstrated progression of the surgical-bed lesions, with additional nodular lesions involving the anterior pelvic peritoneal fascia, left ilio-obturator region, and perineum along the former urethral tract, consistent with locoregional recurrence (**Figure 2B**).

PET-CT in October 2025 confirmed hypermetabolic pelvic disease and revealed intense FDG uptake in the right ventricular mass (SUVmax 14.9; 65x35 mm), with a central hypometabolic component (**Figures 2C****,** **3C**), as well as a small hypermetabolic pulmonary nodule (SUVmax 3.7). Follow-up echocardiography demonstrated further enlargement of the right ventricular mass (52x30 mm), raising suspicion for cardiac metastasis rather than thrombus. In the setting of locoregional and distant progression, enfortumab vedotin (EV) 1.25 mg/kg on days 1,8 and 15 of a 28-day cycle was initiated for metastatic disease in October 2025. Therapy was generally well tolerated, with mild asthenia, pruritus, peripheral neuropathy, and alopecia.

A multidisciplinary board including oncologists, cardiologists, cardiothoracic surgeons, radiologists and nuclear medicine specialists evaluated the case. Cardiac CT (**Figures 3D, E**) and MRI (**Figures 3F, G**) demonstrated a progressively enlarging solid myocardial mass with intracavitary extension involving the mid-apical right ventricle (58 × 53 × 38 mm), arising from the apical septum and ventricular apex. The lesion showed heterogeneous first pass and late gadolinium enhancement on MRI and predominantly peripheral enhancement with a hypodense core on CT (**Figures 3D, E**). It infiltrated the adjacent ventricular septum and free wall, causing systolic restriction and mildly reduced right ventricular function (RVEF, 41%). Given the extent of myocardial infiltration and high risk of ventricular perforation, surgical resection was deemed infeasible.

Molecular profiling of the surgical specimen using the in-house VHIO-300 and VHIO Epsilon DNA/RNA sequencing panels identified a pathogenic *PIK3CA* H1047R variant (variant allele frequency, 15%).

In December the patient referred pelvic pain requiring opioid escalation, and palliative radiotherapy (36 Gy in 12 fractions) was administered providing transient partial symptomatic relief. However, subsequent imaging demonstrated further pelvic and pulmonary progression, and radiotherapy was discontinued (**Figures 2D, E**).

Meanwhile, exertional dyspnea progressively worsened. Although echocardiography in February 2026 showed persistent stability of the ventricular mass and minimal pericardial effusion, the patient’s respiratory symptoms and functional status progressively deteriorated, despite supportive measures, including home oxygen and transfusion support.

In March 2026, following recurrent severe episodes of dyspnea, the patient was admitted to a palliative care unit. He subsequently developed progressive somnolence, hyporexia, pulmonary congestion, and intestinal obstruction, and died seven days after admission. A summary of the case is presented in **Figure 4A** as timeline. Written informed consent for publication was not required, as determined by the institutional ethics committee, because the patient was deceased at the time of manuscript preparation.

**Figure 4:**
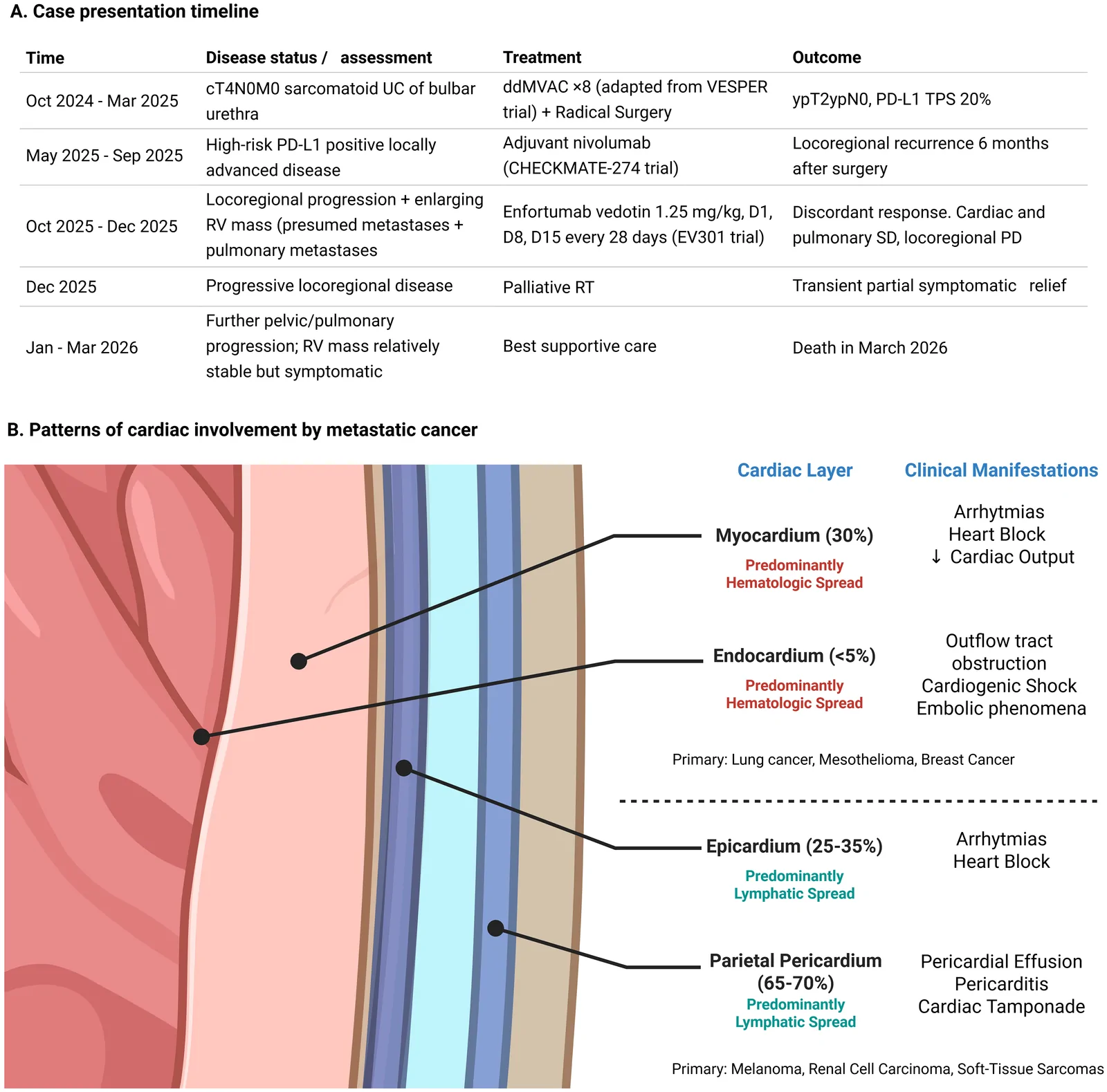
**(A)** Table summarizing the timeline of clinical presentation, therapeutic interventions and outcomes. **(B)** Patterns of cardiac involvement by metastatic cancer according to route of dissemination.

## Discussion

Cardiac metastases may reach the heart through hematogenous or lymphatic spread, with preferential involvement of the myocardium and endocardium through the former and the epicardium and pericardium through the latter. Clinical manifestations range from arrhythmias and conduction abnormalities to heart failure, pericardial effusion, and cardiac tamponade (26, 27) (**Figure 4B**). In our case, the absence of prominent cardiac symptoms despite substantial myocardial involvement until 4 to 5 months after diagnosis, further illustrates the clinically occult nature of these metastases.

Cardiac metastases in the context of urothelial carcinomas with divergent differentiation, including sarcomatoid histology have been previously reported (20–24). Our case therefore adds to an emerging but still limited body of evidence. Its distinctive features include the primary urethral origin, the detailed longitudinal characterization of a right ventricular myocardial lesion using echocardiography, FDG-PET/CT, cardiac CT, and CMR, and the therapeutic decision-making process in a rare, highly aggressive subtype of urothelial carcinoma.

### Diagnostic challenges: cardiac metastasis versus thrombus

A major cardiovascular diagnostic challenge in this case was distinguishing a myocardial tumor with intracavitary extension from an intracavitary thrombus, particularly since the cardiac apex is a recognized echocardiographic blind spot (26). The initial diagnosis of right ventricular thrombus was clinically plausible given the patient’s active malignancy, recent major pelvic surgery and concomitant pulmonary embolism, which provided credible thrombotic substrate. However, the progressive enlargement of the mass despite therapeutic anticoagulation over approximately two months, substantially reduced the likelihood of bland thrombus and prompted further characterization.

The sequential use of multimodality cardiac imaging was particularly informative. Contrast-enhanced transthoracic echocardiography initially characterized the lesion and demonstrated its relationship with the right ventricular cavity and regional wall motion abnormalities. FDG-PET/CT subsequently showed intense metabolic activity within the mass (SUVmax 14.9), providing an important clue toward a malignant process. However, FDG uptake is not specific for malignancy, as metabolically active inflammatory or organized thrombotic lesions may also demonstrate increased uptake (28, 29). In this context, the significance of FDG-PET/CT findings was therefore greatest when interpreted together with the interval growth and the simultaneous progression of pelvic disease. Although the differential diagnosis also included a primary cardiac neoplasm, particularly a primary cardiac sarcoma, the absence of a cardiac lesion on previous imaging and the concurrent extracardiac disease progression made a primary cardiac tumor less likely.

Cardiac CT and cardiac magnetic resonance (CMR) imaging provided key anatomical and tissue-characterization information. Cardiac CT demonstrated a heterogeneous mass with predominantly peripheral contrast enhancement and a central hypodense component. CMR demonstrated that the lesion was not confined to the right ventricular cavity but originated from the apical myocardium, with intracavitary extension and infiltration of the adjacent interventricular septum and right ventricular free wall. The presence of heterogeneous first-pass perfusion and late gadolinium enhancement further supported a vascularized neoplastic lesion rather than bland thrombus and the central non-enhancing component was suggestive of intratumoral necrosis (30–32).

Nevertheless, histological confirmation was not obtained. Cardiac biopsy was not performed due to the extensive involvement of the right ventricular apex and the high perceived risk of myocardial perforation. In addition, progressive extracardiac disease meant that tissue confirmation was unlikely to alter the systemic therapeutic strategy. Although histological confirmation remains the diagnostic reference standard, the risk-benefit balance favored a non-invasive diagnostic approach in this setting. Similarly, surgical resection was considered infeasible because of the extent of myocardial infiltration and the potential risk of ventricular perforation. This emphasizes the importance of multimodality imaging in patients in whom invasive diagnosis or local treatment is not technically or clinically appropriate. No autopsy was performed after the patient’s death, and post-mortem confirmation is no longer possible.

### Therapeutic considerations: neoadjuvant, adjuvant, and metastatic treatment

Because primary urethral carcinoma is rare, prospective evidence guiding systemic treatment is limited, and management is largely extrapolated from urothelial carcinoma of the bladder (30–32). In this patient, treatment of the initial locally advanced disease consisted of neoadjuvant dose-dense methotrexate, vinblastine, doxorubicin, and cisplatin (ddMVAC), followed by surgery and adjuvant nivolumab. In the SWOG-8710 trial, neoadjuvant MVAC, followed by surgery, improved the pathological complete response rate from 15% to 38% and was associated with longer median survival than surgery alone, with survival strongly associated with the absence of residual tumor at surgery (33). Subsequent studies, including a single-arm prospective phase II trial and the phase III VESPER trial, further demonstrated substantial activity of ddMVAC in the perioperative setting and a lower risk of death compared with gemcitabine-cisplatin (34, 35). The subsequent early recurrence should therefore be interpreted in the context of the patient’s initially very high-risk disease, despite substantial pathological downstaging, as the prognostic significance of ypT2 disease appears to be influenced by the initial clinical stage, with patients with cT3/4 disease downstaged to ypT2 having shorter recurrence-free survival than those with cT2 disease who remained ypT2 in retrospective data (36).

Adjuvant nivolumab was administered because of the patient’s high-risk pathological features and PD-L1 positivity. The CheckMate 274 trial demonstrated improved disease-free survival with adjuvant nivolumab in patients with high-risk muscle-invasive urothelial carcinoma after radical surgery (37). Although patients with minor histologic variants were included, the subgroup findings showing a trend towards benefit should be considered hypothesis-generating as prospective evidence specific to histological variants is lacking (37).

At recurrence, enfortumab vedotin (EV) was selected based on the patient’s prior platinum exposure and progression during adjuvant nivolumab, a treatment sequence consistent with the population enrolled in the EV-301 trial, which demonstrated a significant overall survival benefit for EV over chemotherapy (38). Thus, the systemic treatment sequence used in this case was consistent with the available evidence derived from urothelial carcinoma of the bladder and upper urinary tract. The presence of sarcomatoid histology provides additional context for the aggressive clinical course. A meta-analysis of eight observational studies found higher rates of ≥pT3, as well as worse cancer-specific survival (39). Furthermore, a retrospective study evaluating EV found comparable objective response rates between sarcomatoid/other minor subtypes and conventional urothelial carcinoma but significantly shorter progression-free survival in patients with variant histology (40).

The potential role of radiotherapy in this patient also warrants consideration. For initially locally advanced (cT3/T4 N0) primary urethral carcinoma, the EAU Guidelines include surgery followed by adjuvant radiotherapy as one of the multimodal treatment strategies, alongside neoadjuvant chemotherapy followed by surgery and, in selected histologies, chemoradiotherapy (18). However, the guideline does not specifically recommend adding adjuvant radiotherapy after neoadjuvant chemotherapy and subsequent surgery, and the prognostic impact of neoadjuvant and adjuvant treatment modalities remains an area of unmet evidence.

### Molecular insights and future directions

The response pattern observed with EV was characterized by marked interlesional heterogeneity. The patient showed relentless progression of the locoregional disease while the cardiac and pulmonary metastases remained relatively stable during approximately three months of treatment. Organ-specific differences in response to EV have been described in retrospective series, with the highest response rates generally reported in liver metastases and the lowest in bone metastases (41, 42). One potential explanation in our case could relate to the biphasic biology of sarcomatoid urothelial carcinoma. These tumors are thought to arise from conventional urothelial carcinoma through epithelial-to-mesenchymal transition generating a biphasic tumor of common clonal origin (43–45). Because epithelial-to-mesenchymal transition may occur independently in different tumor deposits, metastatic sites may contain different proportions of epithelial and sarcomatoid components (46, 47). Differences in sarcomatoid composition could have therefore contributed to the divergent response observed in this patient, although this hypothesis cannot be directly demonstrated without paired tissue sampling.

Nectin-4 expression represents another potential explanation for interlesional response heterogeneity. Nectin-4 expression may vary between primary and metastatic urothelial carcinoma lesions, and lower expression has been associated with reduced sensitivity to enfortumab vedotin and higher risk of progression in a retrospective cohort (48). Furthermore, lower nectin-4 expression has additionally been described in sarcomatoid histology (49, 50). Nevertheless, Nectin-4 expression was not assessed in our patient. Although archival primary tumor tissue may be available for retrospective testing, no tissue from the cardiac lesion is available, precluding a paired comparison between the primary tumor and the presumed cardiac metastasis. This hypothesis should be considered exploratory and requires validation in studies incorporating paired primary and metastatic tissue.

Molecular profiling of the primary tumor identified a pathogenic *PIK3CA* H1047R variant. Although *PIK3CA* alterations have been associated with prognosis in some cohorts of urothelial carcinoma treated with immune checkpoint inhibitors, their prognostic significance has not been consistently demonstrated across treatment settings (51, 52). In addition, the *TP53/PIK3CA/ATM* risk classifier described in bladder cancer has not been validated in primary urethral carcinoma or in sarcomatoid histology (52). Activation of the PI3K-AKT-mTOR pathway has been linked to a less immunogenic tumor microenvironment, and in bladder cancer, pharmacological PI3K inhibition has been shown to restore anti-PD-1 sensitivity in a *PIK3CA*-mutant model (53). However, *PIK3CA* status does not currently guide treatment selection in the management of bladder or urethral carcinoma, and no clinical trial targeting the PI3K pathway was available at our institution. Thus, although the finding may provide a potential avenue for future investigation, it had no established therapeutic implication at the time of treatment.

## Data Availability

The original contributions presented in the study are included in the article/Supplementary Material, further inquiries can be directed to the corresponding author.
